# The synergic effect of glycyrrhizic acid and low frequency electromagnetic field on angiogenesis in chick chorioallantoic membrane

**Published:** 2015

**Authors:** Shokat Majidian Eydgahi, Javad Baharara, Saeideh Zafar Balanezhad, Majid Asadi Samani

**Affiliations:** 1 *Research Center for Animal Development, Applied Biology & Biology Department, Mashhad Branch, Islamic Azad University, Mashhad, Iran*; 2 *Student's Research Committee, Shahrekord University of Medical Sciences, Shahrekord, Iran*

**Keywords:** *Angiogenesis*, *Glycyrrhizic acid Electromagnetic field*, *Chick Chorioallantoic membrane*

## Abstract

**Objective:**

Much attention is paid to angiogenesis due to its mutual role in health and disease. Therefore, the effect of various chemical and physical agents on inhibition of this process has been recently studied. This study was conducted to investigate the synergic effect of glycyrrhizic acid and electromagnetic field on angiogenesis.

**Materials and Methods:**

In this experimental study, 44 Ross fertilized chicken eggs were randomly divided into four groups, one control and three experimental. Control group was kept with dimethyl sulfoxide on the eighth day, experimental group 1 treated with 200 gauss, 50 Hz electromagnetic field on the 10th day, experimental group 2 treated with 1 mg/ml glycyrrhizic acid on the eighth day, and experimental group 3 simultaneously treated with glycyrrhizic acid on the eighth day and electromagnetic field on the 10th day. On the 12th day, the images of chorioallantoic membrane samples were prepared using photostreomicroscope and the number and length of vessels were measured.

**Results:**

The mean number of vessels in the experimental groups 1 and 3 (29.31±3.60 and 27.43±4.61, respectively) was not significantly different from that in the control group (29.11±4.76) (p>0.05). The length of vessels in the experimental groups 1 and 3 (52.35±3.25 mm and 54.94±4.70 mm, respectively) decreased significantly (p<0.05) compared with the control group (61.79±6.46 mm). In experimental group 2, both length and number of vessels (54.53±5.85 mm and 23.96±3.94) decreased significantly compared with the control group (p<0.05).

**Conclusion:**

Electromagnetic field and glycyrrhizic acid separately led to inhibition of angiogenesis. However, use of electromagnetic field accompanied with glycyrrhizic acid not only did not increase but also decreased the inhibitory effect.

## Introduction

The first blood vessels arise from endothelial progenitor cells with a special arrangement throughout vasculogenesis and gradually began to spread, develop, and establish new branches, which is called angiogenesis (Zafar-Balanezhad et al., 2009 ▶). Angiogenesis is a complex process that requires coordinate activities of various vascular components such as the division of vascular endothelial cells, degradation of basement membrane, and migration of vascular endothelial cells (Hoff abd Machado, 2012 ▶). 

To control this process, several proangiogenic and antiangiogenic factors should interact with each other, including growth factors, angiopoietins, connective molecules, oxygen sensors, and endothelial sensors, among which vascular endothelial growth factor plays a crucial role in angiogenesis and external stimuli such as tissue hypoxia, low blood sugar, and mechanical stress lead to activation of very neatly vascular networks (Ferrara, 2009 ▶; Carmeliet, 2005 ▶). Proangiogenic molecules are kept dormant till angiogenesis is needed for physiological processes such as reproduction, embryogenesis, differentiation of organs, and tissue repair. However, if the balance between proangiogenic and antiangiogenic factors and angiogenic proteins of tumor and host is disturbed, network of angiogenesis will go out of order and the growth continues (Folkman, 2003 ▶; Chung et al., 2010 ▶).

This leads to awakening dormant tumors, and increasing their growth and release into the surrounding tissues. Therefore, it is possible to stop tumor development through discontinuing angiogenesis. As a result, use of drugs that control angiogenesis is a common method of treating these diseases (Baharara et al., 2010 ▶).

 The increased resistance to conventional therapies has become a challenging issue, so that new anticancer agents are needed to promote susceptibility in cancer cells (Kummalue, 2005 ▶). Several studies have indicated that the natural compounds derived from plants deal specifically with formation of new vessels in tumors, with no considerable toxicity and serious side effects on natural tissues. In addition, numerous studies have demonstrated that effective agents present in these compounds are capable of inhibiting proliferation and growth of tumors via other physiologic pathways such as intracellular signaling pathways (Mohammadi-Motlagh et al., 2010 ▶; Mousavi, et al., 2014 ▶; Baharara et al., 2014 ▶). One of the plants which has long been used as medication is liquorice (Glycyrrhiza glabra) from Leguminosae or Fabaceae family. G. glabra is native to Mediterranean region, occurs widely in Southeast Asia, and is important worldwide due to containing medicinal and nutritional compounds in root and rhizome (Nassiri and Hosseinzadeh, 2008 ▶). G. glabra contains various effective compounds, out of which glycyrrhizic acid or glycyrrhizin (chemical formula: C42H62O16) is the most important saponin and one of the most well-known compounds. Glycyrrhizic acid is 50 times sweeter than sugar and has a wide range of pharmacological effects (Khan Ahmady et al., 2013 ▶; Gu et al., 2002 ▶; Asadi-Samani et al., 2015 ▶). 

By numerous studies, glycyrrhizic acid has anti-inflammatory, antiviral, neurally protecting, antitumor, antioxidant, and immune enhancing properties (Kim et al., 2013 ▶), and a high, chemically protective capacity against tumorigenesis (Khan et al., 2013 ▶).

In addition, glycyrrhizic acid could be a significant medicine for treating cancers due to apoptotic effects on tumor cells (Kim et al., 2013 ▶). On the other hand, the introduction and use of more effective therapies are nowadays the interest of researchers and experts. One of these methods is low frequency electromagnetic fields because these fields have become a constant part of human life both naturally and as a result of technology (Baharara et al., 2010 ▶; Ghanati et al., 2006 ▶). Cells stimulation by weak or strong electrical or electromagnetic field can contribute to cell proliferation, ion transport, activation of many enzymes, and increasing intracellular levels of some proteins. These fields also affect the cell membrane permeability by affecting membrane electrical charges (Haj Dezfoulian et al., 2006 ▶). 

Therefore, a biochemical, electrochemical synergism has been already observed which could be used for developing more efficient therapeutic approaches (Bare, 2004 ▶). To answer the question of whether combination of chemical and physical factors affecting angiogenesis has a better outcome for inhibition of angiogenesis or not, this study aimed to investigate the synergic effect of glycyrrhizic acid and 50 Hz, 200-gauss electromagnetic field on angiogenesis. If this field has the capacity to boost antiangiogenic effect of glycyrrhizic acid, it can be used as a supplementary therapy to decrease the dose and hence side effects of chemical compounds.

## Materials and Methods

This experimental, in vitro study was conducted at Animal Development Research Laboratory of Department of Biology, Islamic Azad University, Mashhad Branch in 2012. In this study, we examined angiogenesis in chick chorioallantoic membrane, which is known, among others, as a very suitable in vivo model for investigation of angiogenesis.

Since chorioallantoic membrane begin to form from the fifth day of incubation and occupies more than half of the area inside the egg on the eighth day, and also the heart is completely formed and artery–vein separation occurs on the eighth day, the vascular network was investigated on the eighth day, and imaging and measurements were done on the 12th day (Mousavi et al., 2013 ▶). Forty-four Ross fertilized chicken eggs purchased from Fariman Poultry Complex were randomly divided into four groups of 11 each, as follows: control group was kept with dimethyl sulfoxide (DMSO) (Merck, Germany) on the eighth day, experimental group 1 treated with 200 gauss, 50 Hz electromagnetic field on the 10th day (Baharara et al., 2010 ▶), experimental group 2 treated with 1 mg/ml glycyrrhizic acid (Sigma Alderich, USA) on the eighth day (Kim et al., 2013 ▶), and experimental group 3 simultaneously treated with glycyrrhizic acid on the eighth day and electromagnetic field on the 10th day.

In this study, glycyrrhizic acid powder (molecular weight 822.9, chemical formula: C_42_H_62_O_16_, Sigma Alderich, USA) was used to prepare glycyrrhizic acid solution. To derive the required concentration of glycyrrhizic acid (1 mg/ml), we dissolved 0.01 g glycyrrhizic acid powder in 10 cc DMSO (Haj Dezfoulian et al, 2006 ▶)**.**

In this study, fertilized eggs were placed in a research hatching machine (R.com, South Korea) at automatic rotation, 38 °C and 57% relative humidity. On the second day of incubation, a part of the eggs shell was removed in sterile conditions by laminar hood (Telstar AV-100, Spain) and a window was opened on one side of the egg by the sterile coverslip and paraffin (Arman Sina, Iran).

The eggs were then returned to the incubator and rotated manually twice a day for normal development of the fetus. On the eighth day of incubation, the windows were removed in sterile conditions and a gelatin sponge (containing egg albumin and agar solution in normal saline at equal volume, and 200 µl penicillin streptomycin kept fresh under sterile conditions; 1×4×4 mm^3^) was placed on the chick chorioallantoic membranes. The gelatin sponge was 

already soaked with 10 µl DMSO for the control group and 10 µl glycyrrhizic acid solution (1 mg/ml) for the experimental groups 2 and 3. Then, the window was covered again and the eggs were returned to the incubator. On the 10th day of incubation, the eggs in the experimental groups 1 and 3 were treated with 200-gauss, 50 Hz electromagnetic field inside the hatching machine for 4 h. On the 12th day of incubation, some images of the area around gelatin sponge were taken by a research photostreoscope (Ziess, Germany) with a specific magnification.

**Figure 1:**
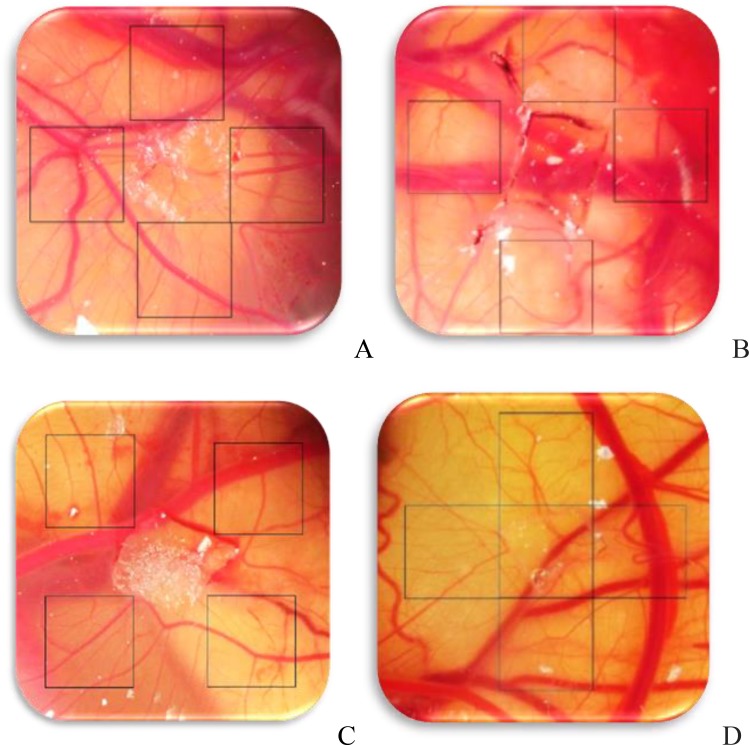
Photostreomicroscopic images of chick chorioallantoic membrane in treated and control samples (80× magnification). The location of sponge has been specified in the images; the squares around the sponges denote the location of counting. A: Control sample (treated with **dimethyl sulfoxide**), B: Experimental group 1 (treated with 200 gauss, 50 Hz electrom agnetic field), C: Experimental group 2 (treated with 1 mg/ml glycyrrhizic acid), and D: Experimental group 3 (treated with glycyrrhizic acid and electromagnetic field).

The images were studied by Image J software, and the number and length of blood vessels were measured in the same cross-sectional area (squares with dimensions 2.5×2.5 cm^2^ on the 4 sides of the gelatin sponge) (Figure 1). The quantitative data were analyzed by SPSS software using ANOVA and Tukey’s test and p<0.05 was considered significant.

## Results

**Figure 2:**
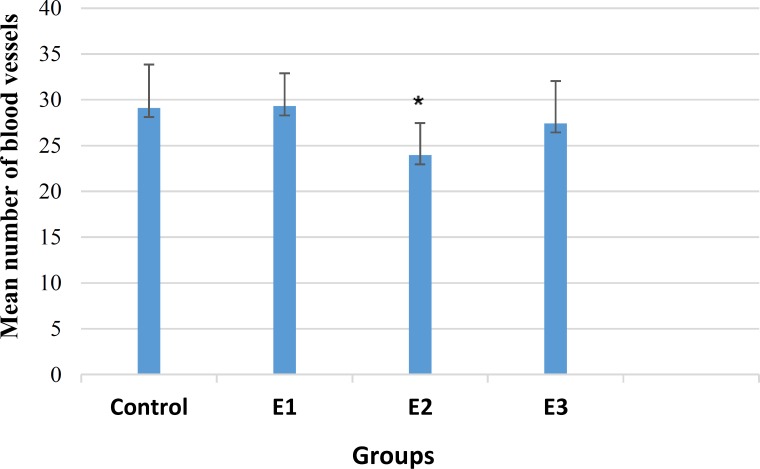
Mean number of blood vessels in treated and control samples; *P < 0.05 compared with the control group; E1: Experimental group 1 (treated with 200 gauss, 50 Hz field), E2: Experimental group 2 (treated with 1 mg/ml glycyrrhizic acid), and E3: Experimental group 3 (treated with glycyrrhizic acid and electromagnetic field).

The mean number of blood vessels in the experimental groups 1 (29.31±3.60) and 3 (27.43±4.61) was not significantly different from the control group (29.11±4.76) (p>0.05, Figure2).

**Figure 3:**
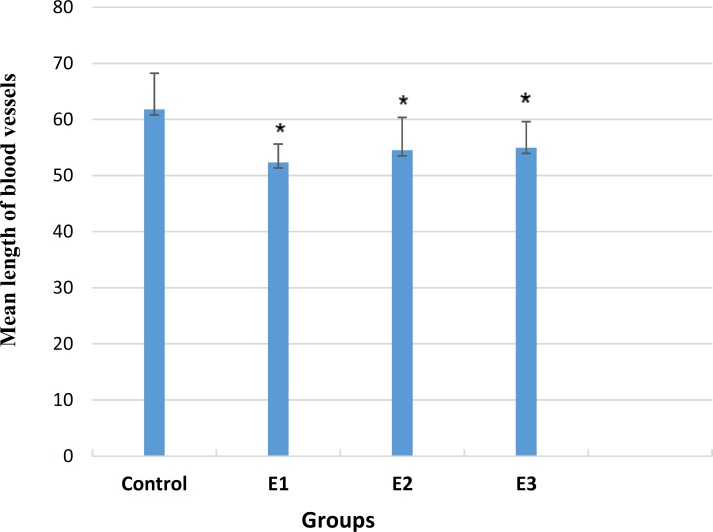
Mean length of blood vessels in treated and control samples; * P < 0.05 compared with the control group; E1: Experimental group 1 (treated with 200 gauss, 50 Hz field), E2: Experimental group 2 (treated with 1 mg/ml glycyrrhizic acid), and E3: Experimental group 3 (treated with glycyrrhizic acid and electromagnetic field).

The mean length of blood vessels was obtained 52.35±3.25 mm and 54.94±4.70 mm in the experimental groups 1 and 3, respectively, with a significant decrease compared with the control group (61.79±6.46 mm) (p>0.05). However, the experimental group 2 exhibited a significant decrease in both the number (23.96±3.49) and length (54.53±5.85mm) of vessels (p<0.05, Figures 2 and 3).

## Discussion

The findings of the present study indicated that glycyrrhizic acid has a wide range of pharmacologic effects (Gu et al., 2002 ▶). When alone, glycyrrhizic acid can inhibit sprouting of new vessels and also prevents the spread of existing vessels as it caused a decrease in number and length of blood vessels in treated groups compared with the control group. Nonetheless, since the effect of magnetic static and electromagnetic fields on the living systems is nowadays notable because of growing application in medicine, industry, and life, we attempted to investigate the combined treatment with glycyrrhizic acid and the field using a low frequency, 200-gauss electromagnetic field. The findings indicated a partial reduction in antiangiogenic effects of glycyrrhizic acid under 200-gauss electromagnetic field.

The antiangiogenic activity of glycyrrhizic acid that led to inhibiting angiogenesis in different steps could be explained by ROS-ERK signalling pathway, which is a crucial cellular event to induce angiogenesis (Kim et al., 2013 ▶). In addition, glycyrrhizic acid has been found as an inhibitor of high mobility group box 1 protein that is used in anticancer and antiangiogenic treatments (Smolarczyk et al., 2012 ▶).

However, there are inconsistent findings with regard to electromagnetic fields’ effect on angiogenic activities that could be attributed to frequency, intensity, and type of the electromagnetic field (Baharara et al., 2010 ▶). For example, William et al. studied the effect of 10, 15, and 20-mT fields on tumor growth and angiogenesis in breast tumor in mice. Their findings indicated that these fields, particularly in the range of 15 to 20 mT, led to considerable inhibition of tumor growth and angiogenesis. The inhibition of angiogenesis was likely to lead to decreased growth of tumor (Williams et al., 2001 ▶).

These findings are consistent with our study with regard to inhibition of angiogenesis, as in the present study 200-gauss electromagnetic field caused a significant decrease in the number of blood vessels compared with the control group. The effects of electromagnetic field on angiogenesis intensification have been studied, as well. For example, Tepper et al. (2009) ▶ found that pulsed electromagnetic field caused a seven-fold increase in tubulization and three-fold increase in endothelial cell proliferation, both of which are considered as important processes in angiogenesis. 

These intensifications were found as associated with increase in secreted angiogenic proteins such as fibroblast growth factor β-2 (FGF-2) (Tepper et al., 2006 ▶). In another study, sinus electromagnetic field with very low frequency and 1-mT intensity increased tubulization and proliferation of umbilical vein endothelial cell (Delle Monache et al., 2008 ▶). 

Another study investigated the effect of pulsed electromagnetic field on lower limbs anemia, a major complication of diabetes which is caused by weak formation of new blood vessels, and found that treatment with these fields could intensify angiogenesis in anemic lesions, and that PEMF caused perfusion and angiogenesis through increasing FGF-2 and activating ERK1/2 pathway in rats with diabetes (Pan et al., 2013 ▶). 

However, in explaining the inhibitory effect of glycyrrhizic acid on electromagnetic field-treated angiogenesis, Bare’s theory on pulsed field to help chemotherapy, through which he created a biochemical, electrochemical synergism for developing more efficient therapeutic approaches, could be helpful. Bare argued that the use of a low frequency, pulsed electromagnetic field could change the physiology and electrochemistry of cancer cells, and influence cell membrane systems and mitosis. In addition, an external field induces some changes in membrane transport capacity through impacting the osmotic potential, ionic valves and endocytic effects of these membranes. These effects on ionic and molecular transitions lead to violation of cellular stress factors, intervention in angiogenesis, increase in the rate of DNA transcription, development of immune response, and inhibition of drug resistance mechanisms (Bare, 2004 ▶).
